# Hypnotism at Berlin

**Published:** 1888-12

**Authors:** 


HYPNOTISM AT BERLIN.—At a late meeting of the Berlin Medical Society, Prof. Virchow introduced a French physician, Dr. Feldmann, who made some experiments in hypnotism. A young man named Garrick offered himself as a medium. After a few seconds of the usual manipulations the medium fell into a deep magnetic sleep. He became perfectly apathetic and motionless. In the state of "suggestion," Dr. Feldmann showed the influence of various medicaments on the medium, who took quinine for sugar, smacking his lips with enjoyment, and he believed ammonia to be perfume, and smelt at it for some time. Immediately afterward, following the will of the doctor, he showed the usual signs of abhorrence of those bitter and caustic substances. With the same success he ate a lemon for an apple. A piece of camphor held on his forehead had a singular effect. The medium bent his body far backward, and had to be held on his chair. A magnet caused a dreamy state, during which the medium related his impressions as to events in the streets in which he believed himself to be. Then the medium obeyed the will of the doctor in various ways, shoveling snow, skating, falling, and rising again with one jump at the doctor's suggestion and finally took a pocket-book by force out of Prof. Virchow's pockets. He was then ordered by Dr. Feldman to reseat himself, and soon woke out of the hypnotic sleep, remembering nothing of what had happened. Two young physicians then spoke, declaring that such experiments were without scientific basis. They believed the 'suggestion" to be probably genuine, but as to the other experiments, especially the effect of medicines and the magnet, they thought they needed careful examination.

